# The 50% and 95% effective dose of remimazolam tosilate for anaesthesia induction in sleep disorders patients undergoing laparoscopic cholecystectomy: an up-and-down sequential allocation trial

**DOI:** 10.1186/s12871-024-02427-7

**Published:** 2024-02-02

**Authors:** Yue Xiao, Yanan Cao, Jie Pu, Chendong Guo, Yanzi Yi, Youming Deng, Yimin Hu

**Affiliations:** 1 Department of Anaesthesiology, The Affiliated Changzhou No. 2 People’s Hospital of Nanjing Medical University, Changzhou, 213003 Jiangsu China; 2 Department of Anaesthesiology, The Affiliated Changzhou No.2 People’s Hospital of Bengbu Medical College, Bengbu, 233000 Anhui China; 3 Department of Anaesthesiology, The Second Hospital of Nanjing, Affiliated to Nanjing University of Chinese Medicine, Nanjing, 210003 Jiangsu China

**Keywords:** Remimazolam, Sleep disorders, Anesthesia induction, Up-and-down sequential allocation trial

## Abstract

**Purpose:**

Previous reports argue that preoperative sleep conditions of patients can influence the dosage of general anaesthesia drugs. Therefore, we aimed to investigate the dose-effect relationship of preoperative sleep disorders on the induction of general anaesthesia with remimazolam tosilate and calculate the Median effective (ED50) and 95% effective (ED95) dosages.

**Methods:**

Included in our study were 56 patients who underwent laparoscopic cholecystectomy at our hospital. A separate group of 27 patients with sleep disorders (SD group) and 29 patients without sleep disorders (NSD group) using the Pittsburgh Sleep Quality Index (PSQI) were also included. According to the Dixon ‘up-and-down’ design, patients received remimazolam at preselected concentrations starting at 0.2 mg/kg. After the administration of remimazolam, loss of consciousness was observed. By observing whether consciousness disappeared within a minute, we adjusted the dose of remimazolam by 0.1 mg/kg (up and down) in the following patient. The Median effective dose (ED50), 95% effective dose (ED95), and 95% confidence interval (CI) of remimazolam for effective sedation were calculated.

**Results:**

The ED50 of remimazolam was 0.226 mg/kg (95%CI 0.221–0.232 mg/kg) in the SD group and 0.191 mg/kg (95%CI, 0.183–0.199 mg/kg) in the NSD group. The ED95 of remimazolam was 0.237 mg/kg (95%CI 0.231–0.262 mg/kg) in the SD group and 0.209 mg/kg (95%CI 0.200–0.254 mg/kg) in the NSD group.

**Conclusions:**

In the SD group, the ED50 and ED95 of remimazolam during anaesthesia induction were 0.226 and 0.237 mg/kg, respectively. The induction dose of remimazolam in the SD group was significantly higher than that in the NSD group.

## Key Points

**Question**: Does the preoperative variation in sleep quality of patients affect the dosage of anesthetic drugs?

**Findings**: Compared to patients with good sleep quality, patients with sleep disorders will require increased doses of the sedative drug midazolam during anesthesia induction.

**Meaning**: Preoperative sleep quality can affect the dosage of drugs during anesthesia induction.

## Introduction

Sleep disorder refers to a state of sleep-wake rhythm disorder mainly manifested by decreased sleep quality [1]. Sleep disorders manifest in abnormal sleep rhythms, such as excessive sleep or insomnia, which can lead to daytime sleepiness and lack of energy. With the rapid pace of social life and increasing work pressure, the proportion of patients with sleep disorders is increasing.

Studies have shown that hypnotic drug effects seem to share a common mechanism and pathway with physiological sleep processes. Narcotic drugs have also been shown to affect the sleep-wake pathway [2] and patients with sleep disorders may require higher doses of narcotic drugs when undergoing general anaesthesia [3–5].

Remimazolam is a new water-soluble ultra-short-acting benzodiazepine class drug that acts mainly on gamma-aminobutyric acid A (GABA-A) receptors, causing reduced body activity, sedation, and amnesia [6]. Remimazolam has been proven to be safe for the induction and maintenance of anaesthesia. It is unknown whether sleep disorders affect remimazolam dosage for the induction of general anaesthesia. Therefore, this study aimed to investigate the dose-effect relationship between remimazolam and loss of consciousness during the induction of general anaesthesia in patients with sleep disorders before surgery and provide a reference for further exploration of the influence of remimazolam on the sleep quality of patients after general anaesthesia.

## Methods and methods

### Ethics

This study was approved by the Medical Ethics Committee of The Affiliated Changzhou No.2 People’s Hospital of Nanjing Medical University ([2022]YLJSA006) on 15 April 2022 and written informed consent was obtained from all subjects participating in the trial. The trial was registered prior to patient enrollment at Clinicaltrials.gov. (10/03/2023, ChiCTR2300069254).

### Study design and participants

We enrolled 56 patients who underwent elective laparoscopic cholecystectomy in our hospital from April 2022 to January 2023. And written informed consent was obtained from 56 patients. All participants were between 18 and 64 yrs and classified according to the American Society of Anesthesiologists physical status classification as I or II, and their body mass index (BMI) was between 18 kg/m^2^−30 kg/m^2^. Patients were excluded if they met any of these criteria: could not cooperate to complete the Pittsburgh Sleep Quality Index (PSQI) score one day before surgery; the presence of severe diseases of the heart, brain, liver, and kidney; a history of mental illness; a complicated or compromised airway; Mallampati grade ≥ II; and participation in other drug or device clinical trials within 3 months before the screening period. All enrolled patients were assessed for sleep performance 1 month before surgery by a trained anaesthesiologist based on the PSQI. Patients were diagnosed as with or without sleep disorders according to the PSQI, in which PSQI > 7 was classified as sleep disorders and PSQI ≤ 7 was classified as non-sleep disorders. Accordingly, patients were divided into a sleep disorders group (SD group, PSQI > 7, *n* = 27) and a non-sleep disorders group (NSD group, PSQI ≤ 7 points, *n* = 29). On the day before surgery, we obtained informed consent from all enrolled patients.

**Fig. 1:**
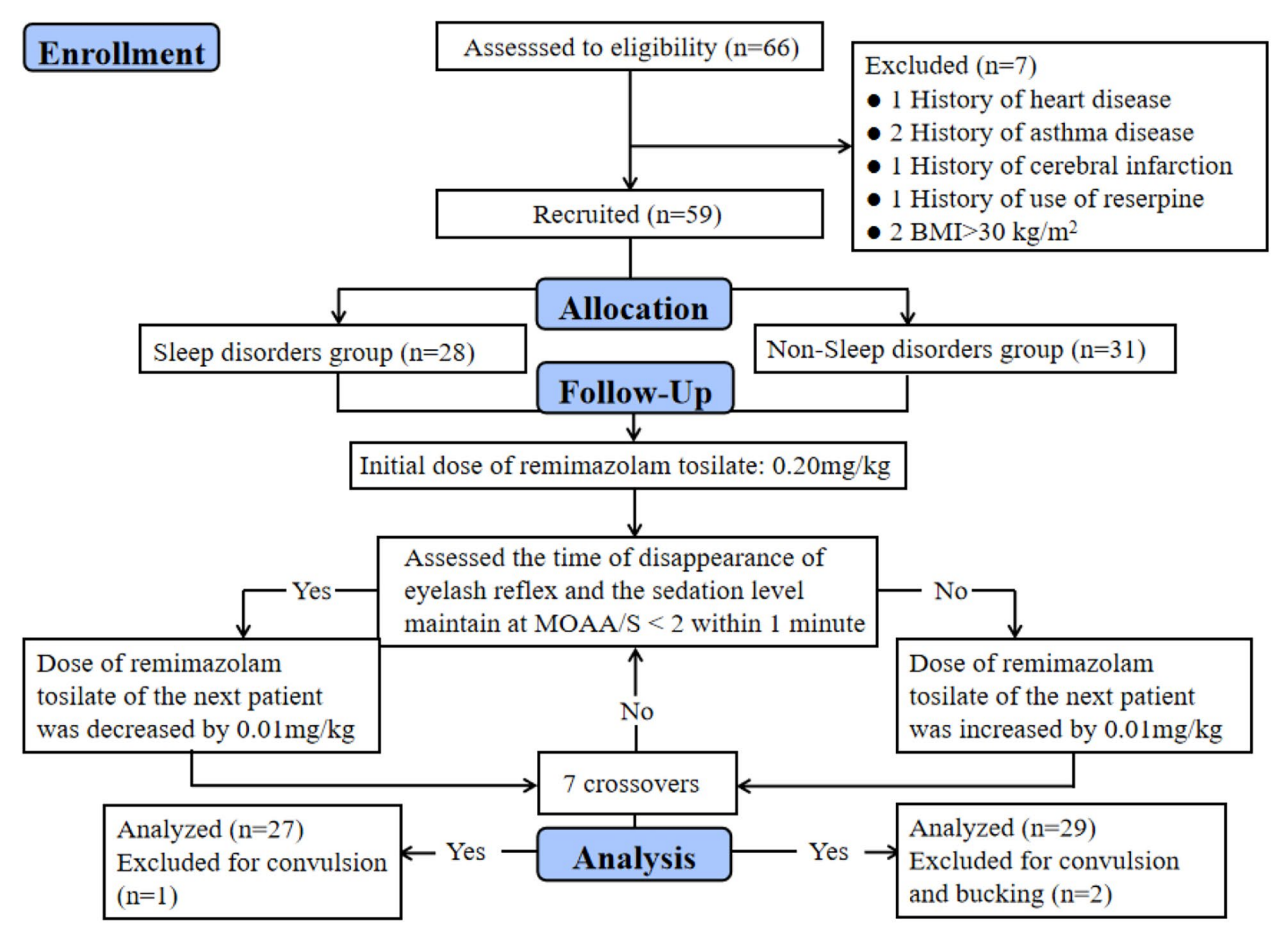
Flow diagram for the Dixon up-and-down method. Seven patients were excluded from observation

### Up-and-down method

Dixon ‘up-and-down’ method was a classical method to determine the efective dose of drugs [7, 8]. The ED95 of remimazolam toluene sulfate for injection was determined using the modified “Dixon ‘up-and-down’ method”. The induced dose for the next patient was determined based on the time to loss of consciousness of the previous patient under a pre-set remimazolam-induced dose. The time to loss of consciousness was determined according to the disappearance of the eyelash reflex and a MOAA/S score < 2 [9]. Eyelash reflex tests and the MOAA/S scale scores were performed by a researcher.

The initial induction dose of remimazolam was “0.2 mg/kg” [10]; the injection was completed within 30 s, and then the duration until the disappearance of the eyelash reflex and a “MOAA/S” score < 2 points were observed. If the eyelash reflex disappeared within 1 min and the MOAA/S score was < 2 points, it was considered positive. If the patient’s eyelash reflex disappeared and MOAA/S score was < 2 points after more than 1 min, it was recorded as negative, and 0.2 mg/kg propofol was added as a remedial measure. If the previous patient had a positive reaction, the next patient received a dose reduced by 0.01 mg/kg. If the previous patient had a negative reaction, the dose was increased by 0.01 mg/kg in the following patient. There were 29 patients without sleep disorders and 27 with sleep disorders were included in the study.

The time to loss of consciousness and reaction results of each patient were recorded. One negative and one positive result in succession were recorded as one cross. The test was terminated after seven crosses were achieved.

### Anaesthesia procedure

Patients fasted from solid food for 8 h and 4 h from clear fluids before anaesthesia and did not receive any sedation, analgesia, or other related drugs for preoperative treatment. After the patient entered the room, the patient’s information was confirmed by three parties, and an intravenous access was used. Ringer’s sodium lactate solution was routinely infused. Standard monitoring was conducted throughout the observation period, including electrocardiogram monitoring, heart rate (HR), non-invasive blood pressure, and oxygen saturation measurements for each patient. Simultaneously, the disposable non-invasive EEG-sensors (BIS: Medlinket) was connected to the brain. And then preoxygenation and denitrogenation for 3 min (oxygen flow rate: 6 L/min).

Anaesthesia was induced by sequential intravenou s infusion of remimazolam toluene sulfate (batch No. H20190034) in 30 s intervals. Eyelash reflexes and modified observer’s assessment alert/sedation (MOAA/S) scores were assessed. After confirming that the patient has reached a certain level of sedation depth ( BIS ≤ 60), administer sufentanil 0.6μg/kg, rocuronium bromide 0.8 mg/kg, and perform tracheal intubation one minute later.

During anaesthesia patients whose developed bradycardia were treated by atropine 0.3–0.5 mg intravenously, and when the mean arterial blood pressure (MAP) < 65 mmHg or 20% of baseline value occurs, intravenous ephedrine 3–6 mg was administered repeatedly if necessary. After the operation, the patient was resuscitated in the post-anaesthesia care unit (PACU) and returned to the ward 30 min later.

### Outcome indicator

Primary outcome measures: The time to loss of consciousness and response results were recorded for each patient, as well as the corresponding remimazolam induction dose and BIS < 60. Secondary outcome indicators: PSQI score, HR, MAP, and SpO_2_ at the time of entry (T0), loss of consciousness (T1), and immediately after the end of intubation (T3). Follow-up was conducted after the patient was awake and whether there was intraoperative awareness during the surgery. Related adverse events and treatment measures during the tests were recorded simultaneously.

### Statistical analysis

SPSS software version 26.0 was used for analysis in this study, and *P* < 0.05 was considered statistically significant. The measurement variables are represented as mean ± standard deviation (SD), and countable data are represented by case number or percentage. The t-test of independent samples was used for pairwise comparisons between groups, and the analysis of variance of repeated measurement data was used for intra- and inter-group comparisons at different time points.

Probit regression analysis was used to calculate the ED50 and ED95 of remimazolam-induced subliminal disappearance in both groups. A 95% confidence interval (CI) was used to calculate the relative median titre.

## Results

All 56 patients completed the study, as shown in Fig. 1. One patient with a history of heart disease, two with a history of asthma, one with a history of reserpine, one with a history of cerebral infarction, two with obesity, two with convulsions, and one with a choking cough were excluded. Finally, 29 patients without sleep disorders and 27 with sleep disorders were included in the study. There was no significant difference in the general data between the two groups (*P* > 0.05; see Table 1).

**Table 1:** Subject characteristics

Characteristic	Patients, No.(%)		
	Sleep disorders (*n* = 27)	Non-sleep disorders (*n* = 29)	P
Sex (male/female)	11/16	10/19	0.863
Age (median, y)	47.1 ± 9.4	49.5 ± 6.8	0.086
BMI (median, kg/m^2)^	24.3 ± 2.1	25.4 ± 2.0	0.899
PSQI scores	8.19 ± 1.08	2.90 ± 1.42	
ASA Physical Status Classification			0.865
I	6(22.2)	7(24.1)	
II	21(77.8)	22(75.9)	
Co-morbidities (n %)			
Hypertension	8(29.6)	9(31.0)	0.909
Diabetes	2(7.4)	3(10.3)	0.535
Hyperthyreosis	0	0	-
History of operation (n %)	2(7.4)	4(13.8)	0.734
Drinking status (n %)	10(37.0)	10(34.5)	0.842
Smoking status (n %)	9(33.3)	8(27.6)	0.430

In four cases, suspected airway obstruction or spasm, severe and violent cough, patient agitation, or lack of cooperation occurred during induction. In such cases, the experiment was terminated immediately for safety reasons, or tracheal intubation was performed as soon as possible after further anaesthesia to control breathing, and these cases are excluded.

**Fig. 2:**
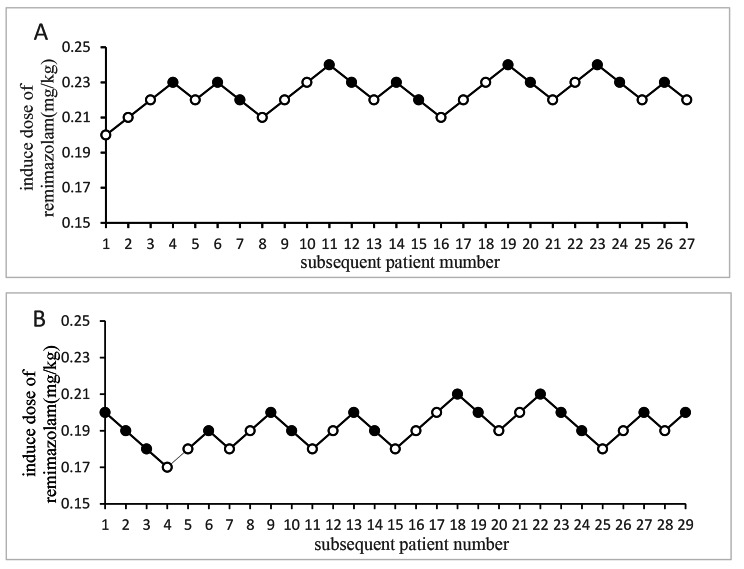
Sequential experimental diagram of Remimazolam for induction of general anaesthesia. **A**: sleep disordered group, **B**: non-sleep disordered group; ‘●’ represent positive reaction, ‘◯’ represent negative reaction

**Fig. 3:**
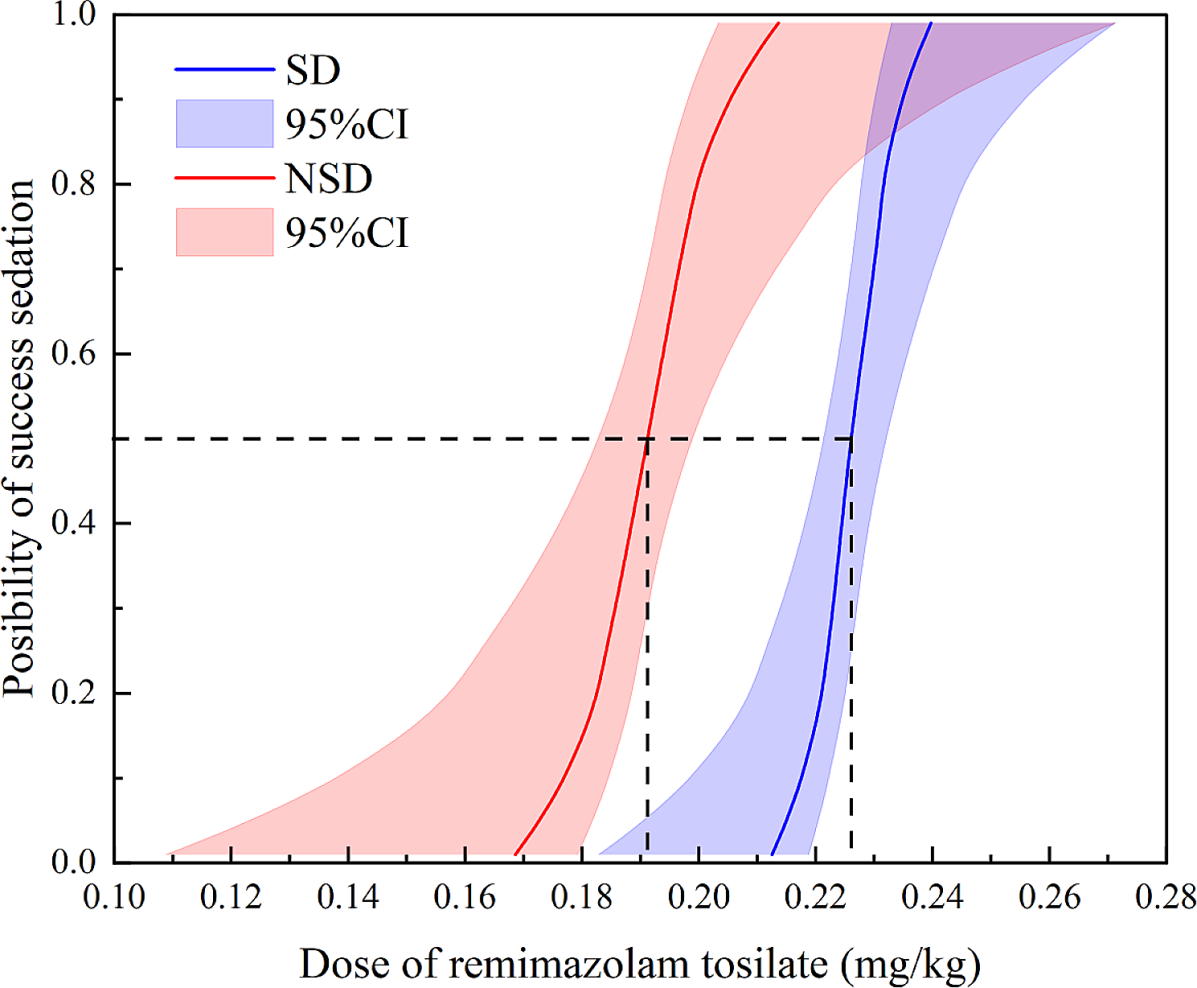
The dose-response curve from the probit analysis of remimazolam dosage and probability of success sedation. Half effective dose (ED50) in the SD group was 0.226 mg/kg (95%CI 0.221–0.232 mg/kg); ED50 in the NSD group was 0.191 mg/kg (95%CI 0.183–0.199 mg/kg)

The dose-response was estimated using the probit model, and according to the dose-response table, the ED50 of remimazolam in the sleep disorders group was 0.226 mg/kg (95%CI 0.221–0.232 mg/kg). ED95 was 0.237 mg/kg (95%CI 0.231–0.262 mg/kg). The ED50 and ED95 values of remimazolam in the non-sleep disorders group were 0.191 mg/kg (95%CI 0.183–0.199 mg/kg) and 0.209 mg/kg (95%CI 0.200–0.254 mg/kg), respectively (see Table 2. and Fig. 2). There was a difference in the median potency between the two groups because the CI did not include one. Figure 3 depicts the dose-response curve of the probability of successful sedation between the two groups.

**Table 2:** Effective Dose of Remimazolam

Parameters	Sleep disorders	Non-sleep disorders	*p*
ED50 and 95%CI	0.226(0.221 ~ 0.232)	0.191(0.183 ~ 0.199)	0.001
ED95 and 95%CI	0.237(0.231 ~ 0.262)	0.209(0.200 ~ 0.254)	0.001

No adverse reactions, such as hypoxaemia, respiratory depression, bradycardia, nausea, or vomiting, were observed in the patients during induction and intubation. Two patients had diaphragm spasms reactions, one had bucking and one had choking and coughing reactions (see Table 3).

**Table 3:** Adverse reactions

Adverse Reactions	Sleep disorders	Non-sleep disorders
Normal adverse reactions		
hypoxaemia	0	0
bradycardia	0	0
nausea or vomiting	0	0
choking and coughing	0	1
Others (Excluded)		
diaphragm spasms	1	1
bucking	0	1

## Discussion

Our study showed that there is a significant difference in the induction dosage of remimazolam between the SD group and the NSD group. In the SD group, the ED50 and ED95 of remimazolam during anaesthesia induction were 0.226 and 0.237 mg/kg, respectively, which were higher than that in the NSD group.

As an indispensable part of human life activities, sleep is a physiological phenomenon that is actively generated and regulated by the brain. It is involved in growth and development, as well as the maintenance of cardiovascular, immune, and cognitive functions, and is especially crucial for the storage and maintenance of memory [11]. However, according to statistics from the World Health Organisation, approximately 27% of people worldwide have sleep disorders [12]. In China, the adult insomnia rate is as high as 38.2% [13], and more than 300 million Chinese people have sleep disorders. Sleep disorders refer to various functional disorders of the sleep-wake process, including sleep initiation and maintenance disorders, insomnia with various causes, excessive sleepiness, sleep-disordered breathing, circadian rhythm, and awakening disorders [14]. Preoperative sleep disorders can lead to peri-operative mental tension, decreased immune function, and hemodynamic instability in patients, thus affecting anaesthesia and surgical outcomes [15], which is not conducive to wound healing, increases hospital stay, and seriously affects prognosis.

The PSQI scale organically combines the quality and quantity of sleep [16] and can effectively, qualitatively, and quantitatively evaluate the sleep condition during the past month. Owing to its high correlation with polysomnography, it is one of the most widely used scales for comprehensively evaluating sleep quality in clinical patients. Studies have shown that the sensitivity and specificity of a PSQI score of 7 as the cut-off point to assess sleep disorders were 98.3% and 90.2%, respectively [17]. Therefore, in this experiment, a PSQI score ≥ 7 was used as the criterion to determine the existence of sleep disorders before surgery.

ED50 and ED95 accurately reflect the dose-effect relationship of drugs. The modified Dixon sequential method adopted in this study is commonly used to calculate ED50 and ED95 [18]. After the initial dose was selected, the dosage of a case was determined based on the response of the previous case. This method is simple, effective, and only requires a small sample size, making it a classic method for clinical studies of drug dose-effect curves [19]. The sequential design requires the initial dose to be selected with the best sedation effec [20]. In this study, the first dose of remimazolam was 0.20 mg/kg based on the experimental results of relevant literature [10] and the pre-test results.

The initiation and maintenance of sleep is an active process that exerts inhibitory control over the ascending awakening nucleus, mainly through the inhibition of GABA in the hypothalamus and basal forebrain [21]. The sleep centre is excited, activates sleep-related active neurones, releases GABA, and promotes the transition from awakening to sleep [6]. Clinical anaesthetic sedative drugs (such as propofol) mainly enhance inhibitory receptors (GABA receptors, glycine receptors) or inhibit excitatory receptors to inhibit the central nervous system and produce a hypnotic effect [20]. Studies have shown that lack of sleep affects the function of the nervous system and thus modulates the impact of general anaesthesia [22].

Previous studies have shown that preoperative sleep disorders can significantly increase the amount of propofol administered during general anaesthesia [23], and postoperative dexmedetomidine can improve patients’ sleep problems, improve sleep quality, and reduce the occurrence of postoperative delirium [24]. Among sedative and hypnotic drugs, benzodiazepines are safe and effective and can enhance sleep quality [25]. Midazolam is a representative drug of the benzodiazepine class with excellent sedative effects and anterograde amnesic properties, and is commonly used for anesthesia induction assistance. However, its half-life of 1.8–6.4 h may lead to a relatively slow recovery from anesthesia after use, requiring patients a longer time to recover from a sedated state. Remimazolam is a new ultra-short-acting benzodiazepine sedative drug, a derivative of midazolam, and another product under the “soft drug” concept after remifentanil [26].Compared with propofol’s injection pain, respiratory depression, and significant hemodynamic effects, adrenal cortical function suppression caused by etomidate, and hypotension and bradycardia caused by dexmedetomidine, remimazolam has the characteristics of fast onset, rapid recovery, no injection pain, and less impact on liver and kidney function and hemodynamics. In addition, it has specific antagonist drugs. These advantages have gradually expanded its clinical application. It is not only suitable for induction and maintenance of anesthesia in the operating room, but also for sedation outside the operating room. It can improve anesthesia effect and promote postoperative recovery, relieve postoperative pain, and reduce adverse reactions.

Remimazolam acts on GABA-A receptors by binding to proteins and promoting the binding of GABA to the GABA-A receptor after binding to the benzodiazepine binding site [27]. This promotes interaction with the associated chloride ion channel, causing a conformational change, leading to hyperpolarization, inhibiting multi-synaptic pathways and causing central nervous system inhibition, thereby exerting sedative/anesthetic effects [28, 29]. Previous studies have found that the blood concentration of remimazolam reaches its peak within 1 min [30]. Theoretically, the prescription of remimazolam is related to the dose, and the higher the dose, the shorter the time for patients to reach a BIS ≤ 60 [32].

The results of this study proved that an increase in the remimazolam induction dose gradually shortened the time to loss of consciousness in patients. However, it reached a peak within a specific range. Subsequently, with the increase in dose, the time to loss of consciousness was not significantly shortened. Previous studies have shown that sleep disorders are related to low GABA and high glutamate levels in the parietal-occipital cortex [32]. In patients with sleep disorders, the compensatory increase in GABA-A receptors requires additional GABA to occupy the corresponding receptors to achieve the ideal effect. Therefore, more remimazolam is needed to achieve an ideal sedative effect during the induction of general anaesthesia, which is consistent with our experimental results.

Previous research on the impact of sleep conditions on the use of propofol indicates that patients with sleep disorders require a higher amount of propofol during general anesthesia to achieve the desired anesthetic effect [33]. And Cao [3] also indicates an increase in the MACawake(minimum alveolar concentration) of sevoflurane in patients with sleep disorders, suggesting a decrease in the sedative efficacy of sevoflurane in this population. Both consistent with the findings of our research.

With the prevalence of the ERAS (Enhanced Recovery After Surgery) concept [34], there is an increased emphasis on personalized medication, tailoring more precise treatment plans based on the individual circumstances of each patient. By considering the patient’s sleep condition, adjusting the drug dosages during the anesthesia process accordingly, and minimizing instances of excessive or insufficient anesthesia, the patient’s hemodynamics can be more stable, leading to a more consistent and optimal anesthetic effect. This approach aims to accelerate postoperative recovery, reduce postoperative hospital stay and enhance postoperative patient quality of life. Meanwhile, maximizing the positive effects of general anesthetics on sleep architecture, optimizing sleep cycles to achieve a stable sleep state, thereby enhancing the postoperative sleep quality of patients, which helps reduce the incidence of postoperative sleep disorders and improves postoperative cognitive function disturbances in patients.

In this study, we also observed a phenomenon of respiratory muscle twitching (upper abdominal twitching) in both groups of patients, but the duration was only a few seconds. After administering propofol, the twitching disappeared immediately and the procedure continued without any significant decrease in oxygen saturation or fluctuations in blood pressure and heart rate. This reaction has not been reported in previous studies, so we speculate that the following possibilities may have caused this: adverse drug reactions of remimazolam; individual differences in patients after using the drug; improper assistance with breathing after anesthesia induction resulting in airway obstruction. It is hoped that this discovery will lead to more research in the future to explore this issue.

This study had some limitations. The PSQI scale was used to evaluate sleep quality before surgery. Although it is a highly relevant to assess sleep quality [35], there are certain subjective factors in the assessment, and it is difficult to subdivide the causes and influencing factors of sleep disorders before surgery. To overcome this, the preoperative assessment of the subjects was conducted by the same anaesthesiologist who had been professionally trained and was familiar with the scale scoring rules. Second, related blood specimen were not collected during the study, and the relevant mechanisms could not be further analysed and explored.

## Conclusion

The experimental results showed that the effective induction dose of remimazolam in the SD group was higher than that in the NSD group, and the ED50 of remimazolam in the SD group was 0.226 mg/kg (95%CI 0.221–0.232 mg/kg). The ED50 of remimazolam in the NSD group was 0.191 mg/kg (95%CI 0.183–0.199 mg/kg). At this dose, the haemodynamic fluctuation of the patients were slight, the adverse reactions were fewer, and the sedation effects were exact. However, further clinical studies are needed to investigate the impact of sleep disorders on the efficacy of general anaesthesia and the effects of general anaesthesia drugs on postoperative sleep function.

## Data Availability

Upon publication all relevant data will be made available from Yue Xiao by the Email if anybody is interested in our study.

## Abbreviations

ED50: The 50% effective dosages
ED95: The 95% effective dosages
PSQI: The Pittsburgh Sleep Quality Index
CI: Confidence interval
